# Genetic Determinants of Non-Syndromic Enlarged Vestibular Aqueduct: A Review

**DOI:** 10.3390/audiolres11030040

**Published:** 2021-08-28

**Authors:** Sebastian Roesch, Gerd Rasp, Antonio Sarikas, Silvia Dossena

**Affiliations:** 1 Department of Otorhinolaryngology, Head and Neck Surgery, Paracelsus Medical University, 5020 Salzburg, Austria; s.roesch@salk.at (S.R.); g.rasp@salk.at (G.R.); 2 Institute of Pharmacology and Toxicology, Paracelsus Medical University, 5020 Salzburg, Austria; antonio.sarikas@pmu.ac.at

**Keywords:** enlarged vestibular aqueduct, pendrin (*SLC26A4*), hearing loss, genetic testing, DFNB4

## Abstract

Hearing loss is the most common sensorial deficit in humans and one of the most common birth defects. In developed countries, at least 60% of cases of hearing loss are of genetic origin and may arise from pathogenic sequence alterations in one of more than 300 genes known to be involved in the hearing function. Hearing loss of genetic origin is frequently associated with inner ear malformations; of these, the most commonly detected is the enlarged vestibular aqueduct (EVA). EVA may be associated to other cochleovestibular malformations, such as cochlear incomplete partitions, and can be found in syndromic as well as non-syndromic forms of hearing loss. Genes that have been linked to non-syndromic EVA are *SLC26A4*, *GJB2*, *FOXI1*, *KCNJ10*, and *POU3F4*. *SLC26A4* and *FOXI1* are also involved in determining syndromic forms of hearing loss with EVA, which are Pendred syndrome and distal renal tubular acidosis with deafness, respectively. In Caucasian cohorts, approximately 50% of cases of non-syndromic EVA are linked to *SLC26A4* and a large fraction of patients remain undiagnosed, thus providing a strong imperative to further explore the etiology of this condition.

## 1. Introduction

Hearing loss affects at least 1 in 1000 newborns, is a major cause of disability in children, and the most common sensorial deficit in humans [1]. In developed countries, where environmental factors, and especially exposure to infections, are no longer expected to play a fundamental role, more than 60% of cases of hearing loss are of genetic origin [2]. Hereditary hearing losses can be further subdivided in non-syndromic, where hearing impairment is the only symptom—70–75% of cases—and syndromic—the remaining 25–30% of cases, which include additional pathological findings [3,4]. Although the great majority of forms of hereditary hearing loss is monogenic, this condition appears to be genetically extremely heterogeneous and may arise from mutations in one of the more than 300 genes known to be involved in the hearing function [5]. The identification of the specific gene responsible for hearing loss provides a conclusive diagnosis to patients and their families, helps in alleviating parental guilt, may reveal additional family members who could be affected, and is essential for a reliable prognosis, planning of an adequate intervention, and precise genetic counseling. 

Although various applications of next-generation sequencing technologies appear appropriate to detect the causative gene in such a complex scenario, these produce a large amount of data that are difficult to analyze and interpret and are not routinely performed. Therefore, the identification of a pathognomonic feature that can orient genetic analysis is still of the outmost importance. In this review, we focus on the enlarged vestibular aqueduct (EVA), which is the most commonly detected malformation of the inner ear. EVA can present in a non-syndromic form, possibly in association with vestibular dysfunction, or as part of complex syndromes such as Pendred syndrome, distal renal tubular acidosis with deafness syndrome, Waardenburg syndrome, Branchio-Oto-Renal (BOR) syndrome, BOR-like syndromes [6], and branchio-oculo-facial (BOF) syndrome [7]. Waardenburg, BOR, and BOF syndromes are autosomal dominant syndromes where distinct clinical findings can guide the identification of the corresponding causative genes [5]. Pathogenic sequence alterations (mutations) or transcriptional dysregulation of a same gene, *SLC26A4*, can cause non-syndromic EVA as well as EVA in the context of Pendred syndrome and distal renal tubular acidosis with deafness syndrome. In Caucasian cohorts with non-syndromic EVA, the identification of the causative gene can be challenging. Here, we give a precise anatomical definition of EVA and we summarize the current knowledge on the genetic factors determining non-syndromic EVA and those forms of syndromic EVA linked to the *SLC26A4* gene. 

## 2. Anatomy, Embryology, and Physiology of the Inner Ear

### 2.1. Principles

The inner ear is made of a complex system of fluid-filled membranous ducts, the so-called membranous labyrinth, which is confined within a thick bony shell, the osseous labyrinth, located within the petrous part of the temporal bone. The structures that are part of the inner ear are the cochlea, the vestibular organ, and the endolymphatic sac and duct. The vestibular organ, in turn, includes the vestibule, which comprises the saccule and utricle, and the three semicircular canals [8]. 

The inner ear originates from the invagination of the otic placode starting in humans from embryonic day 22 post-fertilization. The otic placodes are thickened patches of the ectoderm located in the head region and are involved in the development of the sensory systems of vision, olfaction, hearing, and balance. Invagination of the otic placode into the mesenchyme gives rise to the otic pit. Towards the end of the fourth week of development, the otic pit detaches from the surface ectoderm and forms the otic vesicle, which is lined with columnar epithelium. Between the fifth and seventh week, a fold separates part of the otic vesicle to form the endolymphatic appendage. Additional folds separate the otic vesicle into a dorsal part, which will originate the utricle and semicircular canals and a ventral part, which will form the saccule and cochlear duct. Soon after that, an indenting groove demarcating a tubular diverticulum on the medial side of the endolymphatic appendage forms. This tubular diverticulum differentiates into the endolymphatic duct and sac and continues to grow until the age of four. The membranous labyrinth becomes fully differentiated by week 25 of life [9,10].

While the cochlea is capable of sound mechanotransduction and therefore realizes the sense of hearing, the vestibular organ detects the linear and angular acceleration and controls the sense of equilibrium. These functions are achieved through the activity of highly specialized cells called hair cells, which are part of sensory structures represented by the organ of Corti in the cochlea, the *maculae* in the saccule and utricle, and the *cristae* in the semicircular canals [11]. 

The endolymphatic sac and duct are non-sensory organs of the inner ear. While the roles of the cochlea and vestibular system have been widely studied and well characterized, the functions of the endolymphatic duct and sac remain somewhat more elusive and less discussed. The main functions historically ascribed to the endolymphatic sac are the regulation of the ion composition, pH, volume and pressure of the endolymph, the immune response of the inner ear, and the elimination of endolymphatic waste products. These functions are achieved by light and dark epithelial cells of the intermediate portion of the sac, which are capable of reabsorbing the endolymph and digesting cellular debris by phagocytosis, respectively [12,13]. The light cells and dark cells classically described by Lundquist roughly correspond to the mitochondria-rich cells and ribosome-rich cells, respectively, which have been described in the rat by electron microscopy [14]. The latter is the nomenclature that is widely accepted and currently used.

The epithelial cells of the endolymphatic sac express a battery of water and ion transporters and channels including several isoforms of aquaporin (AQP) and macromolecules related to Na^+^ reabsorption (the Na^+^–K^+^–2Cl^−^ cotransporter NKCC2, the thiazide-sensitive Na^+^–Cl^−^ cotransporter NCC, and the epithelial Na^+^ channel ENaC), acid/base transport (the H^+^-ATPase, the Na^+^–H^+^ exchanger NHE, the Cl^−^/HCO_3_^−^ exchangers SLC26A4/pendrin and SLC4A2, and intracellular and membrane-bound carbonic anhydrase), as well as Cl^−^ transport (the cystic fibrosis transmembrane conductance regulator ion channel (CFTR)). While specific expression in the mitochondria-rich cells has been shown only for few of these transporters, such as SLC26A4/pendrin, it is likely that mitochondria-rich cells orchestrate the ion, pH, and volume homeostasis of the endolymph in the endolymphatic sac [15]. 

### 2.2. Anatomy of the Vestibular Aqueduct

**Figure 1:**
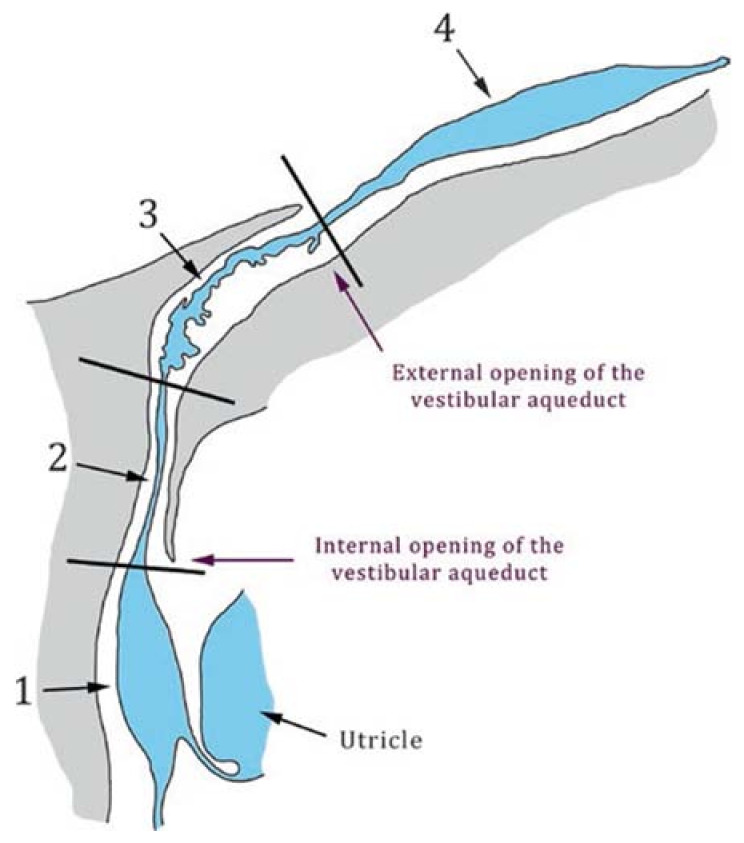
Schematic drawing of the vestibular aqueduct and the endolymphatic duct and sac. The vestibular aqueduct is a bony canal located into the temporal bone (grey) and is delimited by its medial (internal) and external apertures. The endolymphatic duct and sac (blue) are membranous structures that lie within the vestibular aqueduct and comprise the sinus (1), the isthmus (2), a rugose (3), and a smooth portion (4). Black lines indicate the limits between the different portions of the endolymphatic duct and sac. Taken from [16], with permission from Springer International Publishing, 2017.

The vestibular aqueduct (VA) is a bony canal located within the human temporal bone and contains parts of the endolymphatic duct (ED)—the *isthmus* and the rugose portion, which are part of the membranous labyrinth of the inner ear (Figure 1) [16]. The medial, internal aperture opens into the *common crus* of the posterior and superior (anterior) semicircular canals [17], close to the vestibule. From this aperture, the canal runs latero-posteriorly in a J-shaped manner up to its external aperture, also referred to as *operculum*, towards the posterior cranial fossa on the backside of the temporal bone. Along its run within the VA from the medial to lateral sides, the diameter of the ED widens, resulting in a rather narrow, proximal part and a wider, flat distal part. These parts are separated by the narrowest part, the *isthmus* [18]. During embryological development, the initially straight canal is pulled downwards by the sigmoid sinus [19]. Kämpfe Nordström recently provided a detailed overview of the anatomical properties of the VA and the ED [18].

## 3. Malformations of the Inner Ear

### 3.1. Classification

The human ear is classically divided in three portions: the outer, the middle, and the inner ear. In principle, any of these portions can present malformations, which not only correspond to morphological abnormalities, but also lead to functional alterations [20].

The majority of congenital hearing losses (80%) are the consequence of membranous malformations, where pathological changes involve inner ear hair cells. In these cases, diagnostic imaging of the temporal bone reveals normal findings. In the remaining 20% of cases, congenital hearing loss is associated to various malformations involving the bony labyrinth that can be radiologically demonstrated [21].

The identification of the distinct type of malformation is extremely important in that it might present specific therapeutic challenges, dictate the treatment options, and influence their outcome. According to the current literature, a widely accepted classification of the inner ear malformation includes distinct groups, as follows [22,23]:

1. Complete labyrinthine aplasia (Michel deformity): consists of the absence of the cochlea, vestibule, semicircular canals, vestibular and cochlear aqueduct.
2. Cochlear aplasia: consists of the absence of the cochlea.
3. Common cavity: consists of a single chamber representing the cochlea and vestibule.
4. Rudimentary otocyst: represents an anomaly between a Michel deformity and common cavity.
5. Cochlear hypoplasia: the cochlea is smaller than normal, with various internal architecture deformities.
6. Incomplete partition type I (IP-I), or cystic cochleovestibular malformation: the cochlea lacks the entire modiolus and interscalar septa, and therefore has a cystic appearance.
7. Incomplete partition type II (IP-II): the cochlea lacks the apical part of the modiolus. This anomaly was originally described by Carlo Mondini and together with a minimally dilated vestibule and an enlarged vestibular aqueduct (EVA) constitutes the triad of the Mondini deformity.
8. Incomplete partition type III (IP-III): the cochlea has interscalar septa but the modiolus is completely absent. An IP-III is pathognomonic of X-linked deafness DFN3/DFNX2.
9. Enlarged vestibular aqueduct (EVA): this is the most commonly detected malformation of the inner ear [24]. EVA is occasionally associated to other cochleovestibular malformations, such as cochlear incomplete partitions II or III, and can be found in syndromic as well as non-syndromic forms of hearing loss.
10. Cochlear aperture abnormalities: the cochlear aperture transmits the cochlear nerve from the cochlea to the internal auditory canal. An aplastic or hypoplastic cochlear aperture is frequently associated to malfunction of the cochlear nerve.

### 3.2. The Enlarged Vestibular Aqueduct

#### 3.2.1. Definitions of Enlarged Vestibular Aqueduct

Two notations have commonly been used to describe a similar anatomical variant of the vestibular aqueduct—the large vestibular aqueduct (LVA) or enlarged vestibular aqueduct (EVA).

In 1977, Valvassori and Clemis reported on 50 patients with EVA at the 80th Annual Meeting of the American Laryngological, Rhinological and Otological Society. Their subsequent publication in 1978 [25] represents the first definition of a non-physiological extension of the vestibular aqueduct, which has been referred to as EVA or LVA ever since. Based on the radiographic modalities at hand, these authors defined the enlargement as an anteroposterior extension of the bony aqueduct of or greater than 1.5 mm (≥1.5 mm) at the midpoint, visible in axial hypocycloidal polytomography images of fresh post mortem temporal bones. 

In 2007, Vijayasekaran et al. recommended a new definition of potential enlargement, which was based on more precise imaging results acquired through computed tomography imaging (axial planes, 1.0–1.25 mm scans) in 73 children and subsequent definition of normative values for the midpoint and the *operculum* of the VA [26]. These authors concluded to take values of both sites into account for the diagnosis of enlargement and stated that either both or only one of these two measurements exceeding the 95th percentile (>0.9 and >1.9 mm, respectively) justify a diagnosis of enlargement. Moreover, based on radiographic measurements correlated with audiometric findings, Boston et al. confirmed the threshold value of 1.0 mm for the midpoint width of the VA, which was measured in the coronal plane of a computed tomography scan [27]. The criteria of Vijayasekaran and Boston, also referred to as the Cincinnati criteria, identified a larger percentage of pediatric cochlear implant patients compared to the Valvassori and Clemis criterion [28].

Subsequent authors, like Muskett et al. in 2016 [29], only referred to the midpoint threshold value (>1.0 mm) for the identification of EVA.

#### 3.2.2. Diagnostic Tools for Enlarged Vestibular Aqueduct

Given the fact that the object of interest—the VA—is a bony structure, the diagnostic tool of choice for EVA is the computed tomography. However, indirect visualization through the display of the endolymphatic duct by magnetic resonance imaging (MRI) is also feasible and commonly used. Both modalities represent tomography techniques primarily resulting in section images, usually displayed in axial, coronal, and sagittal planes. A normally configured, not enlarged VA is hardly visible in any plane. Owing to the orientation of the VA, as described above, display of the whole VA and subsequent measurement in one single plane does not appear to be possible. The midpoint measurement is commonly done in an axial plane, which displays the greatest extent of the VA; however, Boston et al. recommended performing the midpoint measurement in a coronal plane and measurement at the *operculum* in an axial plane [27]. All authors recommend the use of magnification software tools. There have been further suggestions to use specific, calculated planes for better display [19]. 

Necessity of a reliable and precise definition of enlargement of the VA becomes obvious taking into account the following facts. First, EVA is frequently described as the most common radiological finding in patients with congenital hearing loss [30]. Secondly, as mentioned above, EVA does not necessarily go along with one specific disease. Even though commonly associated with pathogenic variants in the *SLC26A4* gene, EVA has been described in multiple clinical conditions, with and without *SLC26A4* variants. There have been observations of EVA associated with endolymphatic hydrops [31,32] as well as in association with pathogenic variants in genes other than *SLC26A4*. Therefore, the specificity of this radiological finding appears low and needs to be considered as a hint of possible gene alterations and associated pathological conditions rather than a disease-specific feature. 

In order to allow for a clear association of this common radiological sign with any disease, a uniform definition represents a prerequisite for further assumptions on causality and pathogenesis. 

## 4. Genes Involved in Determining Non-Syndromic Enlarged Vestibular Aqueduct

### 4.1. SLC26A4/Pendrin

#### 4.1.1. DFNB4 and Pendred Syndrome

**Figure 2:**
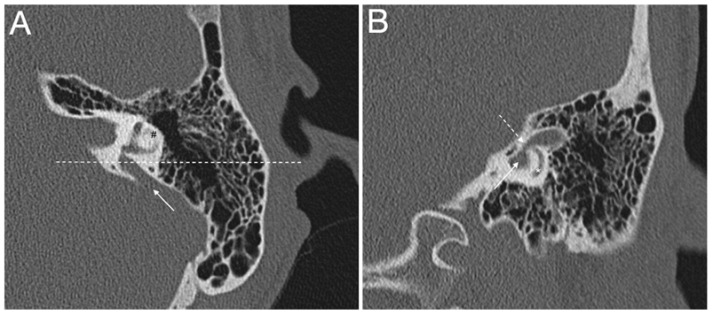
(**A**,**B**) Computed tomography of a left temporal bone of a 12-year-old female patient with bilateral enlarged vestibular aqueduct and congenital moderate (left ear) to profound (right ear) hearing loss. Section A: axial plane; arrow is the enlarged vestibular aqueduct; #, lateral semicircular canal; dotted line is the plane of section B. Section B: coronal plane; continuous arrow is the enlarged vestibular aqueduct; dotted arrow is the isthmus of the enlarged vestibular aqueduct; *, posterior semicircular canal. The patient harbors biallelic pathogenic variants (c.1301C > A; p.A434D and c.1730T > C; p.V577A) in the *SLC26A4* gene, which were described in [43].

**Figure 3:**
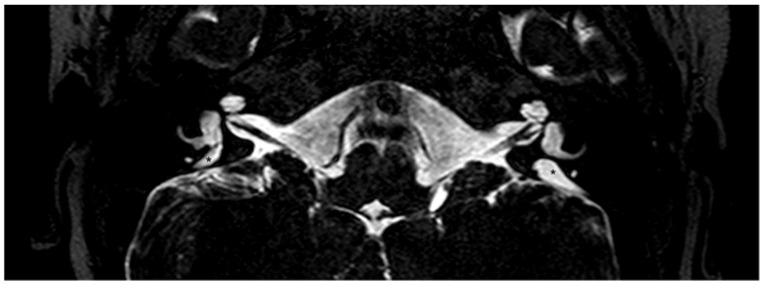
Magnetic resonance imaging of the temporal bones of the same patient shown in Figure 2, T2-weighted. A bilaterally enlarged endolymphatic duct can be observed. *, enlarged endolymphatic duct.

EVA, with or without cochlear incomplete partition II, and accompanied by an enlarged endolymphatic sac and duct, is typically seen in subjects with pathogenic sequence alterations of the *SLC26A4* gene, which codes for the anion exchanger pendrin (OMIM *605646) (Figure 2 and Figure 3). Biallelic mutations in *SLC26A4* lead to Pendred syndrome (OMIM #274600) [33,34] or non-syndromic deafness DFNB4 (OMIM #600791), both of which are inherited in an autosomal recessive manner [5]. In Pendred syndrome/DFNB4, the hearing as well as the thyroid phenotype manifest great variability, often observed also within the same family [35]. In DFNB4, sensorineural hearing loss with or without vestibular dysfunction is the only clinical feature, while in Pendred syndrome, hearing loss is associated to a partial iodide organification defect in the thyroid, which can be disclosed by a positive perchlorate discharge test, with an incompletely penetrant goiter appearing around puberty [36]. Patients are normally euthyroid or subclinical hypothyroid, but overt hypothyroidism may develop [37]. Children with Pendred syndrome/DFNB4 are often born with residual hearing, which is lost around the time of speech acquisition, with a profound impact on language onset and discrimination. In these cases, the degree of hearing loss is moderate to severe and stable. On the other hand, several patients manifest either sudden hearing loss in the adult life, after a head trauma or barotrauma, or fluctuating and progressive hearing loss. In this case, patients receive lifestyle recommendations to maintain residual hearing [38,39]. However, EVA is not thought to be the direct cause of hearing loss in these patients, but indeed represents a radiologic sign of the underlying genetic defect [38,40]. On the other hand, other authors argue that fluid pressure transfer to the cochlea via an EVA can pose a mechanical insult to cochlear structures during embryonic development, possibly leading to bulging of the apex of the cochlea and appearance of an incomplete partition type II, which is actually often seen in association with EVA. High pulsating fluid pressure may be responsible for the progression of hearing loss or sudden hearing loss in these patients [21,23,41]. This topic is currently a matter of debate [42].

#### 4.1.2. The SLC26A4/Pendrin Gene and Protein

The human *SLC26A4*/pendrin gene is located on chromosome 7q31, consists of 21 exons—of which the first is non-coding, and produces a transcript of approximately 5 Kb. In 1997, Everett et al. identified *SLC26A4* as the gene mutated in Pendred syndrome by using a positional cloning strategy [44]. *SLC26A4* is highly homologous to the *SLC26A3*/DRA gene, which is mutated in chloride losing diarrhea (CLD; OMIM 214700). *SLC26A4* is located 3’of *SLC26A3*, from which it probably derives following a duplication event [45].

The SLC26A4/pendrin protein is an electroneutral exchanger for monovalent anions and is expressed in the inner ear [46], thyroid [44], and kidney [47]. In the inner ear pendrin is found on the apical membrane of epithelial cells of the cochlea, endolymphatic sac and duct, and vestibular system. The precise localization of pendrin in these inner ear compartments was firstly described in [48] and reviewed in [49]. Specifically, in the cochlea, pendrin is expressed in epithelial cells of the spiral prominence, outer sulcus cells, root cells, and in spindle-shaped cells of the *stria vascularis* (confirmed in [50]). In the vestibular system, pendrin is expressed in the transitional cells, which are epithelial cells surrounding the sensory structures of the *maculae* and *cupulae*. In the endolymphatic sac, pendrin is expressed in the mitochondria-rich cells. The *stria vascularis* as well as the mitochondria-rich cells are entities controlling the ion and fluid homeostasis of the inner ear. Therefore, the role of pendrin in the inner ear is the control of the ion composition, pH, and volume of the endolymph by secretion of HCO_3_^−^ and reabsorption of Cl^−^ ions, owing to its function as a Cl^−^/HCO_3_^−^ exchanger [51,52]. In the thyroid, pendrin is expressed on the apical membrane of thyrocytes and controls the transport of iodide into the colloid, which is an essential step in the synthesis of thyroid hormones [37]. In the kidney, pendrin is expressed on the apical membrane of beta and non-alpha non-beta intercalated cells of the cortical collecting duct and connecting tubule, and plays a major role in the control of systemic pH and blood pressure by secreting HCO_3_^−^ and reabsorbing Cl^−^ ions [53].

Studies in transgenic mice in which the time of pendrin expression could be experimentally controlled evidenced that, to prevent hearing loss, pendrin is only required during a well-defined time period of embryonic development, i.e., between embryonic (E) day 16.5 and post-natal (P) day 2 [40]. Importantly, reinstatement of pendrin at P6 alleviates fluctuating and progressive hearing loss [54]. These studies led researchers to postulate that interventions during the prenatal or early post-natal phase have the potential of recovering the hearing function in pendrin-linked deafness [49]. Pendrin knockout mice show profound deafness and marked inner ear malformations [49,55] that, however, are not considered the direct cause of hearing loss, which is rather thought to be the result of acidification of the cochlear endolymph, the consequent loss of endocochlear potential, and elevation of endolymphatic Ca^2+^ concentration, ultimately leading to degeneration of sensory hair cells in the organ of Corti [49]. Interestingly, in a new mouse model expressing the common pathogenic pendrin variant p.L236P, inner and outer air cell degeneration is postponed compared to pendrin knockout mice, and the degree of hearing loss is variable from mild to profound [56]. Together, these considerations suggest that a temporal window of possible intervention in patients expressing pathogenic pendrin variants actually exists. Prenatal electroporation of normal *Slc26a4* cDNA to homozygous knockout mice otocysts utilizing biphasic pulses at E11.5 induced pendrin expression, significantly attenuated enlargement of the endolymphatic space, and prevented post-natal hearing loss and vestibular dysfunction [57].

Apart of gene therapy, no mechanistic approaches leading to restoration of hearing in Pendred syndrome/DFNB4 have been developed up to now. Testing of the function of the pendrin variants found in deaf patients revealed that the pathological phenotype is the consequence of a partial or total impairment of pendrin ion transport activity [58]. The main pathomechanism of disease was initially believed to be retention of the misfolded pendrin protein variants within subcellular compartments [59]. Recently, however, we showed that reduction of protein levels, rather than mislocalization, is the key feature of pathogenic pendrin protein variants [43,60]. Accordingly, retention within the endoplasmic reticulum is not seen for all pendrin variants. Indeed, approximately only 50% of pathogenic variants remain trapped within the endoplasmic reticulum and manifest a severe loss of function, while the other variants are correctly localized at the plasma membrane and have significant residual function [43,60]. As mentioned above, we showed that, in heterologous expression systems, the global as well as the plasma membrane protein levels of pathogenic pendrin variants are significantly reduced compared to the wild type, with no alteration in the corresponding mRNA levels [43,60]. Accordingly, in a mouse model expressing the hypomorphic pendrin variant p.S408F [61], the levels of the mutated pendrin protein in the kidney cortex resulted as decreased compared to those of wild-type littermates [62]. Thus, a reduction of protein levels may represent a novel pathomechanism for Pendred syndrome/DFNB4 and targeting the degradation pathway may represent a novel therapeutic strategy. 

#### 4.1.3. Prevalence of *SLC26A4* Sequence Alterations in Cohorts with EVA

The prevalence of *SLC26A4* sequence alterations in EVA cohorts is strongly influenced by patient ethnicity. In cohorts from different regions of China, biallelic mutations in *SLC26A4* were found in 65–95% of EVA patients [63,64,65], while the remaining patients had no mutations (20%, [64]), or had one or no mutations (12% and 23%, respectively, [65]). In more recent studies, virtually all bilateral EVA cases seemed to be diagnosed with biallelic *SLC26A4* mutations (95.54%, [66]), probably reflecting an increased sensitivity of sequencing techniques.

A high prevalence (57–84%) of biallelic *SLC26A4* mutations has similarly been observed in Taiwan [67], Korean [68,69], and Japanese EVA cohorts [70].

**Figure 4:**
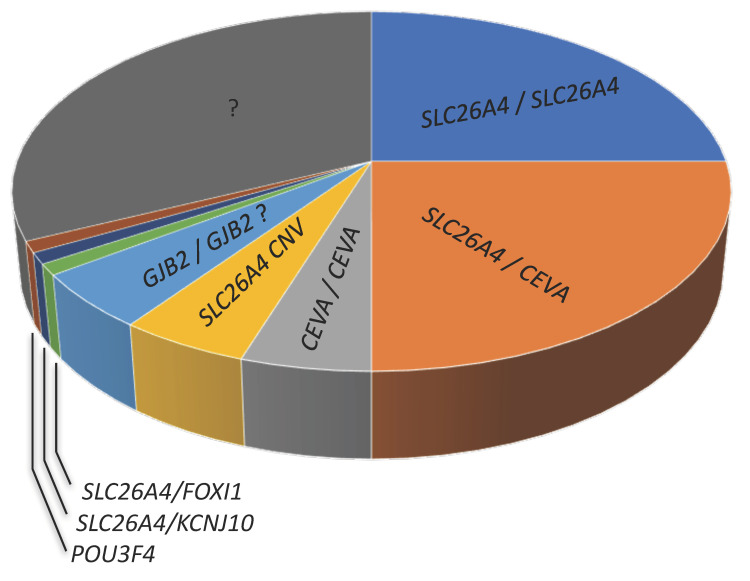
Approximate proportion of genetic determinants (first allele/second allele) of hearing loss and EVA in Caucasian cohorts. CEVA, Caucasian enlarged vestibular aqueduct haplotype; CNV, copy number variation; ? indicates a controversial finding or undetermined feature.

In Caucasian cohorts, the picture outlined above changes substantially. While there is a strong correlation between Pendred syndrome and biallelic (M2) mutations in the pendrin gene [71], biallelic mutations are found only in approximately one-fourth of patients with non-syndromic EVA. Of the remaining EVA patients, approximately one-fourth harbor monoallelic (M1) pendrin mutations (the second mutated allele cannot be identified) and about a half are negative for pendrin mutations (M0) [72,73], (Figure 4 and Table 1). Therefore, a large proportion of EVA patients in Caucasian cohorts remain undiagnosed [43].

The segregation ratio of EVA in M1 families is not significantly different from that seen in M2 families and is consistent with the predicted ratio (25%) for an autosomal recessive trait, thus suggesting the existence of a second, undetected *SLC26A4* mutation in M1 families [74]. The number of mutated alleles correlates with phenotype, and M1 and M0 patients have less severe hearing loss and a lower prevalence of cochlear implantation [72,75].

It has been estimated that *SLC26A4* deletions and duplications represent no more than 1.8–8.7% of the “missing” *SLC26A4* mutations [76,77]. Additionally, in unilateral EVA, mono- or biallelic pendrin mutations seem to be less prevalent (25%) compared to bilateral EVA [78]. Therefore, other genetic factors are expected to play a role in M1 and M0 patients.

**Table 1:** Prevalence of *SLC26A4* mutations in large (*n* > 30 individuals) EVA cohorts with a predominant Caucasian ethnicity. The number and % of patients with biallelic (M2), monoallelic (M1), or no mutations (M0) in the *SLC26A4* gene in a given cohort is indicated.

Probands (*n*)	M2	M1	M0	Reference
58	9 (16%)	14 (24%)	35 (60%)	[79]
39	14 (36%)	14 (36%)	11 (28%)	[80]
100	24 (24%)	16 (16%)	60 (60%)	[81]
429	57 (13%)	75 (17%)	297 (69%)	[82]
474 ^1^	66 (14.5%)	89 (19.5%)	303 (66%)	[83]
83	20 (24%)	16 (19%)	47 (57%)	[75]
85	7 (8%)	17 (20%)	61 (72%)	[84]
123 ^2^	32 (26%)	15 (12%)	76 (62%)	[72]
115	87 (76%)	16 (14%)	12 (10%)	[85]
66	40.2%	17.5%	42.1	[86]
Average	28%	19.5%	52.5%	

^1^ Overall, 125 patients had Mondini dysplasia and 349 had EVA. ^2^ Expansion of the cohort originally described by King et al. [75]. The subjects of this study are cochlear implant recipients.

#### 4.1.4. *SLC26A4* and Meniere Disease

Meniere disease (OMIM 156000) is a clinical spectrum of rare disorders characterized by recurrent vertigo attacks, fluctuating sensorineural hearing loss, and tinnitus, with familial as well as sporadic forms. Endolymphatic hydrops, which is the expansion and bulging of the *scala media* into the *scala vestibuli*, can be considered a histopathological sign of Meniere disease, although cases of asymptomatic endolymphatic hydrops as well as cases of Meniere disease with no endolymphatic hydrops have been reported [87]. It has formerly been suggested that, in some cases, EVA and Meniere disease with endolymphatic hydrops may be linked to a common dysfunction of the fluid homeostasis of the inner ear [88]. Recent findings corroborate this hypothesis. Studies of gene expression profiling identified genes expressed in the *stria vascularis* and involved in controlling the ion and fluid homeostasis of the inner ear as possible pharmacological targets for disorders of hearing and balance, including Meniere disease [50]. Localization of genes implicated in Meniere disease to distinct cell types of the *stria vascularis* denotes that functional alterations of these cell types may be involved in the pathophysiology of Meniere disease [89]. Accordingly, sequence alterations in genes expressed in the *stria vascularis* have been linked to Meniere disease. Specifically, in a large cohort of 890 patients with sporadic Meniere disease, enrichment in rare missense exonic variants in *SLC26A4* and *KCNJ10* genes has been reported when their minor allele frequency (MAF) was compared to reference data from ExAC, non-Finnish European, and Spanish populations [90]. In the *stria vascularis, KCNJ10* is expressed in the intermediate cells, and plays a fundamental role in the maintenance of the endochoclear potential. The role of *KCNJ10* in EVA is discussed in the following section.

### 4.2. Genes and Genetic Factors That Have Been Linked to EVA in Association with Monoallelic Pendrin Mutations

#### 4.2.1. *FOXI1* and *KCNJ10*

Additional genes that have been linked to EVA in association with monoallelic pendrin mutations are *FOXI1* (OMIM *601093), which codes for a transcription factor of *SLC26A4* [91], and *KCNJ10* (OMIM *602208), which codes for an inwardly rectifying potassium channel essential for the maintenance of the endocochlear potential [92].

Biallelic *FOXI1* mutations lead to a complex syndrome called distal renal tubular acidosis with deafness (dRTA; OMIM #267300) in individuals who are negative for the known causative genes, which are *SLC4A1*, coding for a Cl^−^/HCO_3_^−^ exchanger, and *ATP6V0A4* and *ATP6V1B1*, coding for vacuolar proton pumps [93]. This finding is explained by FOXI1 being a transcription factor for the genes causing dRTA as well as for *SLC26A4* [94,95]. *Foxi1* knockout mice are deaf, display signs of vestibular dysfunction, and show a great expansion of the inner ear compartments that becomes evident at E16.5, with the endolymphatic duct and sac as the most expanded structures, in association with the absence of *SLC26A4* expression [91]. Accordingly, one of the probands with biallelic *FOXI1* mutations originally described by Enerbäck et al. showed bilateral EVA [93].

Of note, EVA has been described as part of the phenotypic alterations of dRTA [96] independently from *SLC26A4* mutations but linked to *ATP6V1B1* and *ATP6V0A4* [97,98,99,100,101]. Accordingly, mice bearing a spontaneous mutation in the *Atp6v1b1* gene exhibit profound hearing impairment associated with an enlargement of the endolymphatic compartments of the inner ear [102].

Biallelic mutations in *KCNJ10* cause sensorineural hearing loss as part of a rare and complex syndrome, called EAST/SeSAME syndrome, consisting of epilepsy, ataxia, sensorineural deafness, and renal tubulopathy (OMIM #612780). In *Slc26a4* knockout mice, the *Kcnj10* protein expression is absent in the intermediate cells of the *stria vascularis* and the endocochlear potential is lost [48]. These alterations probably play a fundamental role in determining hearing loss in Pendred syndrome/DFNB4. 

Digenic inheritance of EVA caused by a monoallelic mutation in *SLC26A4* and another monoallelic mutation in *FOXI1* or *KCNJ10* has been suggested as a hypothesis [82,103]. For *FOXI1*, this hypothesis is supported by segregation studies and the observation of an EVA in the *Slc26a4^+/−^; Foxi1^+/−^* mouse mutant. Regarding *KCNJ10*, haploinsufficiency of *Slc26a4* in the *Slc26a4*^+/*−*^ mouse mutant resulted in reduced protein expression of *Kcnj10* in the *stria vascularis* [82,103]. However, the genetic configuration of double heterozygosis is either infrequent [76,104], considered as non-pathogenic [105,106], or not found in Caucasian [107,108], Taiwanese [109], East Chinese [110], Chinese [111,112], and Korean [113] cohorts. Occasionally, monoallelic *FOXI1* sequence variants in the absence of *SLC26A4* mutations are found in patients with EVA [67,114], but the causality of these findings is uncertain. Other studies found biallelic *SLC26A4* mutations in combination with monoallelic *FOXI1* of *KCNJ10* mutations, but segregation studies indicated the biallelic *SLC26A4* mutations as causative and lead to exclusion of the pathogenicity of the double heterozygous genotype [106,115]. Copy number variations in *FOXI1* of *KCNJ10* were not found in an EVA cohort of 46 patients [77]. Overall, *FOXI1* or *KCNJ10* mutations have been estimated to account for approximately 1% of EVA cases [74,76,114].

#### 4.2.2. The Caucasian EVA (CEVA) Haplotype

In Caucasian cohorts, a newly discovered haplotype, called Caucasian EVA (CEVA), seems to be responsible for EVA in the majority of patients with monoallelic pendrin mutations and at least in some patients (approx. 4.8%) with no mutations in known causative genes [116]. These finding arose from massively parallel sequencing of the *SLC26A4* region in EVA patients with one or no mutations in the *SLC26A4* gene belonging to two distinct Caucasian cohorts—a United States EVA discovery cohort and a Danish EVA replication cohort.

The CEVA haplotype consists of 12 single nucleotide polymorphisms (SNPs), including 10 single nucleotide substitutions and 2 single nucleotide deletions, falling in intergenic regions or non-coding genomic regions of other genes, far upstream of the *SLC26A4* gene. It was postulated that the CEVA haplotype might lie within regulatory regions of the *SLC26A4* gene and therefore control the expression of pendrin. However, the ability of the CEVA haplotype to affect expression of the *SLC26A4* gene has not been demonstrated.

The CEVA haplotype correlates with phenotype severity in patients with EVA and seems to play a dual role in this context. The CEVA haplotype *in trans* to a mutation of *SLC26A4* is associated to a normal thyroid phenotype and less severe hearing loss compared to patients harboring biallelic *SLC26A4* mutations. However, in patients with no *SLC26A4* mutations, the CEVA haplotype is associated to a more severe hearing loss. These observations indicate that the CEVA haplotype can act as a pathogenic recessive allele in combination with biallelic *SLC26A4* mutations and as a genetic modifier of EVA linked to factors other than *SLC26A4* [117].

#### 4.2.3. *EPHA2*

The *EPHA2* gene (OMIM *176946) encodes the ephrin receptor EphA2. The Eph (erythropoietin-producing hepatocellular carcinoma) receptors represent the largest family of receptor tyrosine kinases in mammals and play a critical role in a variety of housekeeping cellular functions during development and in mature tissues. The binding of Eph receptors to their plasma membrane-bound ligands results in oligomerization and trans-phosphorylation events that may result in a bidirectional signaling across the plasma membrane, eventually culminating in control of cell adhesion, repulsion, migration, morphology, and proliferation [118].

A recent report proposed a digenic inheritance of DFNB4/Pendred syndrome caused by monoallelic mutations in *SLC26A4* and *EPHA2* [119]. These authors found that *EphA2* knockout mice showed an enlarged cochlear duct and thyroid hypertrophy, which are reminiscent of clinical features of Pendred syndrome. In the inner ear, kidney, and thyroid of these mice, pendrin expression in the cell apical membrane was disrupted, clearly indicating that EphA2 controls pendrin localization. Pendrin established a direct molecular interaction with EphA2 in the inner ear, kidney, and thyroid. Some pathogenic variants of the pendrin protein lost their ability to interact with EPHA2. Sequencing of the *EPHA2* gene in 40 Japanese patients with hearing loss and monoallelic mutations in the *SLC26A4* gene who were negative for the CEVA haplotype led to the identification of two individuals with potentially pathogenic *EPHA2* sequence variants. The corresponding protein variants conserved their ability to bind pendrin; however, following stimulation with the ligand ephrin-B2, but not with ephrin-A1, the EphA2/pendrin complex failed to be internalized. These findings suggest that aberrant regulation of the correct membrane targeting of pendrin via EphA2 may contribute to DFNB4/Pendred syndrome [119].

### 4.3. POU3F4

*POU3F4* (OMIM *300039) is a gene found on the human X chromosome and encodes a transcription factor widely expressed in the neural tube during development, but subsequently restricted to specific structures in the adult forebrain, including the anterior region of the hypothalamus [120].

Mutations in *POU3F4* lead to mixed conductive and sensorineural X-linked DFN3/DFNX2 deafness (OMIM #304400) associated to stapes fixation, cochlear incomplete partition type 3, and perilymphatic gusher during stapedectomy, and can account for up to 0.5% of all cases of severe childhood hearing disorders [121]. *Pou3f4*-deficient mice show a lack of differentiation of otic fibrocytes in the spiral ligament [122] as well as a lack of expression of *Kcnj10* in the intermediate cells of the *stria vascularis* [123]. Interestingly, this latter defect is reminiscent of that seen in *Slc26a4* knockout mice and similarly occurs at the onset of the endocochlear potential generation between P10 and P17 [48,124]. 

A variety of temporal bone anomalies in addition to cochlear incomplete partition type 3, and especially a widening of the internal acoustic canal but no EVA, have been described in patients with *POU3F4* mutations by several authors [125,126,127,128]. In contrast, Choi et al. and Pollak et al. found an EVA in three out of five and four out of eight patients, respectively [129,130].

When present, EVA in these patients seems to be different from that described in DFNB4/Pendred syndrome; Incesulu et al. presented four case reports where vestibular aqueducts showed great inter-patient variability, but all were large and symmetrical and become cystic or enlarged from the middle parts to the ends near the vestibule [131]. Accordingly, Kanno et al. identified 6 patients (0.6%) with incomplete partition type 3 and *POU3F4* mutations among 1004 children with hearing loss; all of them had an enlargement of the vestibular aqueduct close to the vestibule but not at the operculum; these authors linked this specific finding with an absence of hearing fluctuations in these patients [132]. Similarly, Anderson et al. described two brothers with *POU3F4* mutations and dilation of the portion of the vestibular aqueduct close to the vestibule [133].

To conclude, *POU3F4* mutations are rare in EVA patients and do not invariability lead to EVA. In patients with *POU3F4* mutations, EVA has specific anatomical features, is accompanied by other temporal bone deformities, and is seen in approximately 50% of cases. 

### 4.4. GJB2

The *GJB2* gene (OMIM *121011) encodes for Connexin 26, a gap-junction protein classically described as involved in potassium recycling within the cochlea [134], for which more complex functions have been recently proposed [135].

Mutations in *GJB2* alone account for 50% of cases of hereditary deafness in various cohorts and represent the leading cause of hereditary deafness worldwide [136]. Although specific *GJB2* mutations are involved in determining an autosomal dominant form of deafness (DFNA3) as well as various forms of syndromic deafness associated with skin disease; the great majority of mutations in this gene cause non-syndromic autosomal recessive hearing loss DFNB1. This form of sensorineural hearing loss is prelingual in its onset, mild to profound in severity, non-progressive, and it is described as usually not associated with vestibular and/or labyrinthine abnormalities [4]. However, several studies reported isolated EVA cases or EVA cohorts where monoallelic or biallelic *GJB2* mutations were found [137,138,139,140,141]. 

Propst et al. reviewed CT imaging of 53 cochlear implant recipients with biallelic *GJB2* mutations and found that 72% of subjects had at least one temporal bone anomaly and 8% had EVA [142]. In a large retrospective study of 108 children with sensorineural hearing loss and EVA, Lee et al. identified 2 patients with biallelic *GJB2* mutations, although the proportion of patients with EVA and no *GJB2* mutations was significantly higher [143]. Kenna et al. examined 113 patients with biallelic GJB2 mutations and established that 10% had various and subtle abnormalities of the temporal bones, but none had EVA [144]. 

It is difficult to evaluate whether findings of *GJB2* mutations in EVA patients are causative or coincidental. As a matter of fact, in our cohort of Caucasian patients with EVA, 2/33 (6%) harbor biallelic pathogenic sequence alterations in *GJB2*. These patients were negative for other known EVA causative genes [16]. It can be concluded that mutations in *GJB2* are only seen in a minority (0–10%) of EVA patients, and the causative link between EVA and *GJB2* is not unequivocally established. *GJB2* mutations can coincidentally be detected in patients with an EVA determined by other factors. In this context, it is important to exclude other known EVA causative genes, as *GJB2* and *SL26A4* mutations can even coexist in a same patient [145].

### 4.5. TMC1

*TMC1* (OMIM *606706) underlies dominant and recessive non-syndromic hearing loss at the DFNA36 and DFNB7/B11 loci [146] and encodes for a protein expressed in cochlear and vestibular hair cells required for hair cell mechanoelectrical transduction [147]. 

A recent report documented the identification of *TMC1* variant(s) in EVA patients [141]. We recently described a patient showing hearing loss with an autosomal dominant pattern of inheritance and a pathogenic variant in the *TMC1* gene. This patient had an EVA [148]. These findings suggest that *TMC1* mutations might contribute to EVA in patients who are negative for the classical causative genes in the context of dominant deafness or when the pattern of inheritance of deafness cannot be determined.

### 4.6. Other Genetic Factors

Additional genes found mutated in association with EVA in a Chinese cohort were *PCDH15*, *MYO6*, and mitochondrial genes [141]. *PCDH15* (OMIM *605514) encodes for protocadherin 15 and is mutated in Usher syndrome types 1D/F and 1F, as well as in non-syndromic autosomal recessive deafness DFNB23 [149]. *MYO6* (OMIM *600970) encodes for nonconventional myosin VI and is mutated in autosomal dominant deafness DFNA22, with or without hypertrophic cardiomyopathy, and autosomal recessive deafness DFNB37 [150]. 

Next-generation sequencing technologies are revealing an increasing number of rare genes associated with EVA in patients negative for *SLC26A4* mutations; these genes are expected to be heterogeneous, especially in cases on unilateral EVA, and probably strongly correlated to the ethnicity of the cohort [151].

## 5. Conclusions

1. In Caucasian cohorts, *SLC26A4* and/or the CEVA haplotype, *FOXI1*, *KCNJ10*, *POU3F4*, and possibly *GJB2* may account for hearing loss and EVA in approximately 50–65% of patients;
2. The genes that are causative of EVA remain unidentified in 35–50% of patients (Figure 4);
3. The genetic determinants of EVA in undiagnosed patients are probably strongly heterogeneous, especially in cases of unilateral EVA;
4. In undiagnosed patients, causative sequence alterations may fall in regulatory regions or in genes coding for unidentified regulatory factors of the established genes and proteins, in genes not formerly linked to EVA, or even in genes not formerly linked to deafness.

## 6. Future Perspectives

It will be important in future studies to confirm the presence of the CEVA haplotype in additional Caucasian cohorts, and to assess its possible role in regulating *SLC26A4* expression. To better ascertain the pathogenic role of the CEVA haplotype *in trans* with monoallelic pendrin mutations, these should be carefully characterized by functional testing to discriminate between pathogenic and benign sequence variants. Additionally, it would be important to explore the role of *EPHA2* in Caucasian cohorts, and to confirm the double-heterozygous inheritance model with *SLC26A4* by segregation studies.

Next-generation sequencing technologies are predicted to identify an increasing number of novel genes carrying variants in EVA cohorts; assessing whether these findings are causative or coincidental will remain a great challenge. The combination of a precise clinical characterization of the patient’s hearing phenotype and functional studies of the mutated genes and the corresponding proteins will be essential to confirm or exclude a causative link between a given genetic variant and disease.

## Data Availability

Not applicable.
